# Does interpersonal self-support matter for freshman nursing students’ professional identity? Evidence from mainland China

**DOI:** 10.3389/fpsyg.2023.1123625

**Published:** 2023-05-23

**Authors:** Ting Zhang, Dan Su, Yajuan Yang, Shuwen Li

**Affiliations:** School of Nursing, Anhui Medical University, Hefei, China

**Keywords:** nursing, student, China, professional identity, interpersonal self-support, latent profile analysis

## Abstract

**Background:**

Many studies have focused on undergraduate nursing students’ professional identity (PI), but freshman nursing students (FNSs) have been ignored, and the relationship between interpersonal self-support (ISS) and PI is unknown. This study was designed to determine the patterns of ISS and its association with PI among Chinese FNSs.

**Methods:**

A cross-sectional survey was conducted among 358 FNSs recruited from two nursing colleges in southeast China. Students completed the Sociodemographic Characteristics Questionnaire, the Interpersonal Self-Support Scale for Adolescent Students, and the Professional Identity Questionnaire for Nurse Students. Latent profile analysis (LPA) was used to determine the patterns of ISS among freshmen. The Bolck–Croon–Hagenaars method was used to examine the influencing role of ISS in PI.

**Results:**

LPA indicated that ISS could be classified into three subgroups: the ISS-Individualist group (7.54% of the total sample), ISS-Dependent group (63.13% of the total sample), and ISS-Extrovert group (29.33% of the total sample). Overall, these three profiles differed significantly in the five dimensions of ISS and PI (*p* < 0.05). The results of pairwise comparisons examined the positive role of the ISS-Extrovert group on the promotion of PI among FNSs.

**Conclusion:**

These findings emphasize the need for the promotion of PI and ISS among Chinese FNSs. Freshman students need more confidence and general communication knowledge to maintain harmonious social relationships with others. Parent-teacher association could be applied to nursing education to guide FNSs’ positive development of ISS.

## Introduction

The shortage of nursing staff has been a crucial challenge for the promotion of public health worldwide (Xu et al., 2021). For example, China will be 2 million nurses short of achieving its “Health China 2030” target (Chen et al., 2018). It has been widely demonstrated that the high turnover of nurses is responsible for this global problem (Xu et al., 2021). The estimated average rate of turnover is 68% a year among nurses globally (Labrague et al., 2019). There are many influencing factors that contribute to the high turnover, such as multiple care needs, increasing working pressure, low social status, and hazardous working conditions (Van der Heijden et al., 2019). According to the theory of career development constructed by Tiedeman and O’Hara (1963), individuals try to solve the working difficulties they confront by integrating their personality into feasible career goals to reshape their professional identity (PI), and as a result promote their career development. In other words, the patterns of personality and the development of PI facilitates the transformation of vocational behaviors during different career periods (Tiedeman and O’Hara, 1963). PI is a special form of one’s sense of identity defined as the self-cognition of roles, norms, beliefs, and values associated with one’s personal career (Rose et al., 2018). The benign formation of PI is beneficial for both nurses themselves and patients (Rose et al., 2018). Existing empirical research has clarified the crucial effects of PI on nurses’ subjective well-being, psychological reward satisfaction, and willingness to provide support (Li et al., 2022). Moreover, the formation of PI is a dynamic process of socialization involving professional skills, attitudes, and understanding of roles (Rose et al., 2018).

### Formation of professional identity

Professional education and clinical nursing experience are vital for the formation of PI (Fitzgerald and Clukey, 2021). As a result, the level of PI varies in different stages of professional education and the clinical nursing career (Mao et al., 2021). Mao et al. (2021) divided the formation of nursing PI into five stages of pre-formal professional education, formal professional education, under 5 years of nursing practice, 5–20 years of nursing practice, and more than 20 years of nursing practice. These five stages are shaped by outside professional experience, contradictions between ideals and realities, self-confidence and passion for nursing, balancing needs between family and work, and low morale at work, respectively (Mao et al., 2021). They also summarized the influence factors of PI into four interactional categories: social factors (such as social discrimination, traditional values, and perceived professional images by the public), family factors (such as family support, family income, and occupations of parents), institutional factors (such as salary levels, employment status, and hospital rankings), and personal factors (such as gender, age, and motivations for choosing nursing) (Mao et al., 2021). Compared with other nursing PI theories, this theoretical framework was based on Miller’s theory, which integrated pre-formal professional education into the development of PI (Mao et al., 2021). However, the summarized influence factors of PI overemphasized the external contributors, which may neglect of the effect of personal traits in the interactions between the educational or clinical environment and nursing students or nurses.

PI is the outcome of the development of personal identity in the career context (Rose et al., 2018). Identifying the self is an important aspect of PI. The results of empirical studies have demonstrated the interactional relationship between these two kinds of processes of personal development (McAdams, 1995). Rasmussen et al. (2018) found that registered nurses’ PI consisted of the self, the responsibilities of professional role, and their career environment. Goodolf (2018) described the formation of PI among baccalaureate nursing students as involving self-doubt, confidence, sacrifice, rigor, and relevance. Additionally, personality traits are at the core of the self, and the effects of personality traits on registered nurses’ PI have been demonstrated (McAdams, 1995). Wang and Zhang (2017) examined the close relationship between PI and extraversion and neuroticism from the Big Five personality traits among Chinese nurses. It has been widely accepted in Western counties that personality traits are universal across environments and cultures (Chien, 2016). However, as cross-cultural studies of personality traits become more intensive, several indigenous Chinese personality traits conceptualized using Chinese traditional culture are emerging, such as the seven-factor Chinese personality, the self-support personality, good and evil personality traits, and the thick black personality (Zhou et al., 2009; Tang and Guo, 2015; Chien, 2016; Jiao et al., 2021; Yao et al., 2022). It is widely believed that these indigenous Chinese personality traits might be more suitable for exploring the relationship between personality and personal development among Chinese people (Chien, 2016). To date, studies focusing on the effect of these indigenous personality traits on nursing PI are limited. Furthermore, because of the social features of PI, the concept, influencing factors, and pathways shaping nursing PI were found to be inconsistent in different social contexts and nursing education systems (Fitzgerald and Clukey, 2021). Relevant studies concentrated on the relationship between PI and personality traits among nursing students has so far been limited.

For nursing students, guiding the positive process of PI development is also crucial for enhancing the constancy of nursing teams (Browne et al., 2018). In addition, nursing students construct their PI even before obtaining professional education, potentially forming an unrealistic image of the nursing profession according to the social value of nurses among many students (Browne et al., 2018). In China, nurses have always been treated as having low levels of education, a high-pressure working environment, and low social status (Zhang et al., 2021). As a result, the nursing profession is not the first choice of many Chinese freshman nursing students (FNSs), but it is often chosen due to course allocation in colleges being based on student preferences (generally, nursing courses are the least preferred by students) or based on others’ recommendation (Wang et al., 2022). Many students switch to other medical majors in their sophomore year or relinquish their nursing professional education in the senior year (Wang et al., 2022). Therefore, the first year of nursing education is an important period for cultivating positive PI to facilitate the retention of undergraduate nursing students (Browne et al., 2018). However, prior research has focused on the overall undergraduate nursing population sample, graduate nursing students, and first-year student nurses, while FNSs were ignored (Fitzgerald and Clukey, 2021; Qi et al., 2021; Zhang et al., 2021). Furthermore, college life is a crucial period for shaping personality (Yao et al., 2022). Applying personality traits and PI to nursing research among freshmen is beneficial for the promotion of the retention of the nursing group and for positive personal development.

Goodolf (2018) summarized the formation of PI among baccalaureate nursing students as the process of continually searching for balance and utilizing support networks, which consisted of social support, faculty support, and academic support. Freshmen especially need to adapt to the new learning environment, new interpersonal relationships, and new challenges, and social support from relatives and friends and academic support are essential for them to adapt to the nursing profession (Goodolf, 2018). For senior nursing students, social support from nursing peers, faculty support, and academic support were vital for them to adapt to unanticipated events (Goodolf, 2018). Therefore, for both freshmen with pre-professional education and senior nursing students with professional theoretical education and clinical nursing rotation training, interpersonal communication with relatives, friends, teachers, and mentors are crucial for them to find support to cultivate their PI (Goodolf, 2018). In China, interpersonal communication with others not only represents individual social competence but also reflects an interpersonal trait that helps indigenous Chinese deal with interpersonal problems and develop harmonious social relationships (Xia et al., 2015). This indigenous personality trait is called interpersonal self-support (ISS) (Xia et al., 2015).

### Interpersonal self-support

ISS is a dimension of the self-supporting personality that incorporates interpersonal communication with a set of Chinese traditional virtues including independence, initiative, responsibility, flexibility, and openness (Yao et al., 2022). The self-supporting personality is considered a positive personal trait that protects individuals from problems across the lifespan (Yao et al., 2022). As the social part of the self-supporting personality, ISS contains five personality traits–interpersonal independence, interpersonal initiative, interpersonal responsibility, interpersonal flexibility, and interpersonal openness–and it is beneficial for solving daily interpersonal problems and promoting individual social development (Yao et al., 2022). Clearly, ISS concerns both interpersonal independence and connection, which is different from traditional Western personality traits (Yao et al., 2022). Researchers have found that ISS may be influenced by familial, peer, and personal factors, such as education status of parents, parenting style, parent–child relationship, family function, peer relationships, and being an only child (Yao et al., 2022). Evidence from empirical research has supported the positive role of ISS in school belonging, cooperative behavior, social adaption, and depressive symptoms (Yao et al., 2022). Meanwhile, Xia et al. (2012) found that the ISS traits predicted perceived social support. The first year of nursing education is an important period for utilizing social support to adapt to the college life (Goodolf, 2018). Incorporating ISS within the PI may help expand our knowledge with respect to the positive formation of PI and personality among FNSs. However, the research on ISS applied in the nursing profession is limited. The patterns of ISS and its role in PI are unknown.

### Current study

Previous studies on ISS and PI were likely to use variable-centered methods, which aim to identify the level, influencing factors, distal outcome variables, and structural model of PI and ISS without regard for the differences among students. They assumed that there was no heterogeneity among subtypes. According to the preceding overview, this study aimed to describe the patterns of ISS and examine its influencing role in PI among FNSs. As the social part of self-supporting personality, different patterns of ISS may exist among different populations. That is, population diversity should be considered to draw conclusions more in line with the characteristics of FNSs, and to identify more useful implications for nursing education to guide their positive development of well-being. For this reason, the person-centered method may be more appropriate for our study. This method highlighted the sample’s heterogeneity and constructed a typology or clustering of individuals with similar characteristics to classify observed units into latent subgroups (Von Eye and Bergman, 2003). Accordingly, we hypothesized that the ISS of students would be heterogeneous and classified them into several patterns using LPA, which is widely used for person-centered analysis with continuous data (Gabriel et al., 2015). Then we examined the differences in PI between the subgroups of ISS using the Bolck-Croon-Hagenaars (BCH) method. This study is the first to use person-centered analysis to examine the patterns of ISS and the influencing role of ISS in PI among Chinese FNSs. This may deepen the understanding of the formation of PI among Chinese FNSs and provide an empirical reference for designing more effective interventions to promote the positive development of ISS and PI in them.

## Methods

### Design and participants

A cross-sectional survey was employed among Chinese FNSs from May to September of 2021 in two nursing colleges in the Anhui Province of China. FNSs were recruited using convenience sampling. The inclusion criteria were as follows: (1) passed the National College Entrance Examination; (2) was a full-time baccalaureate FNSs; (3) was willing to complete our survey; and (4) had normal comprehension and writing ability. Exclusion criteria included psychiatric disorders, post-associate degree baccalaureate FNSs, undergraduate self-educated FNSs, and refusal to participate. In terms of the LPA sample size, researchers advised a minimum sample ranging from 300 to 1,000 (Xia et al., 2012). In our study, in total of 384 questionnaires were distributed, and 358 were found eligible for data analysis, resulting in a response rate of 93.23%.

### Ethical considerations

Our study was conducted with approval from the university (No. 20210075). The aim, importance, and approach of this study were explained by researchers to all FNSs before they completed the questionnaires. We also explained the principles of voluntary and anonymous participation to all students. Signed consent forms were completed by students. In our study, all the students were not compensated with credits or awards in any way.

### Measures

The Sociodemographic Characteristics Questionnaire was used to collect the demographic and professional information of the FNSs. The collected information included age, gender, being an only child, residence, education status of father, education status of mother, monthly family income, and academic achievement level.

To evaluate the features of ISS among FNSs, we used the Chinese version of Interpersonal Self-Support Scale for Adolescent Students (ISSS-AS), which was developed by Xia and Huang (2009). The whole scale is presented in Supplementary Table S1. This scale includes 20 items across 5 dimensions: interpersonal independence, interpersonal initiative, interpersonal responsibility, interpersonal flexibility, and interpersonal openness (Xia and Huang, 2009). Each item is rated from 1 (completely disagree) to 5 (completely agree; Xia and Huang, 2009). Higher scores represent better ISS status. The scale is widely used in psychological studies in China, with adequate internal consistency (Xia and Huang, 2009). The criterion validity of the scale was found to be adequate in comparison to other Chinese personality scales (Xia and Huang, 2009). In this study, Cronbach’s alpha coefficient was 0.75.

The Chinese version of Professional Identity Questionnaire for Nurse Students (PIQNS) was used to measure the extent of students’ PI (Hao et al., 2014). The whole scale is presented in Supplementary Table S2. This 17-item scale consists of 5 dimensions: professional self-image, benefit of retention and risk of turnover, social comparison and self-reflection, independence of career choice, and social modeling (Hao et al., 2014). Each item is scored from 1 (completely disagree) to 5 (completely agree) (Hao et al., 2014). The internal consistency coefficients was 0.83 (Gabriel et al., 2015). Exploratory principle component factor analysis supported the structure of the scale (Gabriel et al., 2015). In the current study, Cronbach’s alpha coefficient of the whole scale was 0.87.

### Data collection procedure

After obtaining the approval of the ethics committee, two trained researchers contacted the college counselor to find potential participants. Then one researcher called the students to determine the date and time of the session. After explaining the aim, importance, and approach of our study to students at the beginning of the study, the students completed consent forms and then filled out the questionnaires independently with standardized instructions. Students took about 10 min to complete the questionnaires.

### Data analysis

We analyzed the data using IBM SPSS Statistics version 20.0 for Windows and Mplus 7.4. LPA was employed to describe the patterns of ISS. The fit statistics included the Akaike information criterion (AIC), Bayesian information criterion (BIC), sample size-adjusted BIC (a-BIC), entropy, value of *p* on the Lo–Mendell–Rubin likelihood ratio test (LMR-LRT), and bootstrapped likelihood ratio test (BLRT) (Gabriel et al., 2015). The AIC, BIC, and a-BIC should be relatively lower, the entropy value should be higher than 0.08, and the value of *p* on LMR-LRT and BLRT should be significant (Gabriel et al., 2015). Then we used the BCH method to explore the differences in PI on latent profiles of ISS (Gabriel et al., 2015). Shapiro–Wilk tests, basic statistical description, correlation analysis, and univariate analysis were also performed as supplementary analyses. values of *p* < 0.05 were considered statistically significant.

## Results

### Preliminary analysis

Students’ ages ranged from 16 to 21, with a median of 18 and a quartile spacing of 1. Of the students, 83% were female and 76.5% came from rural areas. The specific sociodemographic characteristics of Chinese FNSs are presented in Table 1.

**Table 1:** Comparison of sociodemographic information of freshmen nursing students among three interpersonal self-support (ISS) profiles (*N* = 358).

Category	Subcategory	Total sample	ISS-Individualist group	ISS-Dependent group	ISS-Extrovert group	*χ*^2^	*P*
Gender, *n* (%)	Male	61 (17.0)	4 (14.8)	37 (16.4)	20(19.0)	0.465	0.792
	Female	297 (83.0)	23 (85.2)	189 (83.6)	85 (81.0)		
Age, *n* (%)	<18 years old	43 (12.0)	4 (14.8)	25 (11.1)	14 (13.3)	0.567	0.753
	≥18 years old	315 (88.0)	23 (85.2)	201 (88.9)	91 (86.7)		
Being an only child, *n* (%)	Yes	78 (20.7)	6 (22.2)	54 (23.9)	18 (17.1)	1.921	0.383
	No	280 (74.3)	21 (77.8)	172 (76.1)	87 (82.9)		
Residence, *n* (%)	Urban	84 (23.5)	5 (18.5)	52 (23.0)	27 (25.7)	0.690	0.708
	Rural	274 (76.5)	22 (81.5)	174 (77.0)	78 (74.3)		
Education status of father, *n* (%)	Primary school	66 (18.4)	3(11.1)	43 (19.0)	20 (19.0)	3.938	0.415
	High school	257 (71.8)	23 (85.2)	157 (69.5)	77 (73.3)		
	College or above	35 (9.8)	1 (3.7)	26 (11.5)	8 (7.6)		
Education status of mother, *n* (%)	Primary school	126 (35.2)	8 (29.6)	81 (35.8)	37 (35.2)	1.229	0.873
	High school	217 (60.6)	18 (66.7)	134 (59.3)	65 (61.9)		
	College or above	15 (4.2)	1 (3.7)	11 (4.9)	3 (2.9)		
Monthly family income, RMB, *n* (%)	≤1,000	51 (14.2)	3 (11.1)	28 (12.4)	20 (19.0)	5.085	0.459
	1,001–2,000	85 (23.7)	5 (18.5)	61 (27.0)	19 (18.1)		
	2001–3,000	101 (28.2)	9 (33.3)	61 (27.0)	31 (29.5)		
	≥3,001	121 (33.8)	10 (33.3)	76 (33.6)	35 (33.3)		
Academic achievement level, *n* (%)	Good	194 (54.2)	9 (33.3)	120 (53.1)	65 (61.9)	7.774	0.100
	Medium	147 (41.1)	16 (59.3)	94 (41.6)	37 (35.2)		
	Poor	17 (4.7)	2 (7.4)	12 (5.3)	3 (2.9)		

The results of Shapiro–Wilk tests revealed that the total score of the ISSS-AS and PIQNS showed no significant deviation from normality (*p* > 0.05), and the average level of ISS and PI were 53.40 (9.02) and 71.16 (8.20), respectively. Because of the skewed distribution, the scores of the subscales of ISSS-AS and PIQNS were expressed as “median (interquartile range).” The scores of the subscales of ISSS-AS and PIQNS are shown in Table 2.

**Table 2:** Scores of professional identity and interpersonal self-supporting.

	PSI	BRRT	SCS	ICC	SM	PI	IId	II	IR	IF	IO	ISS
M (P25, P75)	18 [15, 20]	11 [9, 12]	11 [10, 12]	7 [6, 8]	7 [6, 8]	–	13 [11, 15]	13 [11, 15]	16 [15, 18]	14 [13, 16]	15 [13, 16]	–
M/M (SD)	18.49	10.83	11.39	7.89	7.54	53.40 (9.02)	15.46	15.63	17.54	15.95	16.25	71.16 (8.20)
Skewness	−0.224	0.077	−0.253	−0.12	−0.326	−0.063	−0.206	−0.282	−0.554	−0.181	−0.088	0.172
Kurtosis	0.497	0.817	0.639	−0.005	0.028	0.588	−0.255	−0.027	0.357	0.383	−0.34	−0.141
*p*	0.000	0.000	0.000	0.000	0.000	0.066	0.001	0.000	0.000	0.000	0.000	0.177

PSI, Professional self- image; BRRT, Benefit of retention and risk of turnover; SCS, Social comparison and self-reflection; ICC, Independence of career choice; SM, Social modeling; PI, Professional identity; IId, Interpersonal independence; II, Interpersonal initiative; IR, Interpersonal responsibility; IF, Interpersonal flexibility; IO, Interpersonal openness; ISS, Interpersonal self-supporting.

### Latent profile analysis

We employed six models to explore the classification of ISS. Fit statistics are summarized in Table 3. The entropy values of the last four models were acceptable (>0.80), so the second profile was rejected. Then, we compared the values of AIC, BIC, and a-BIC of these six profile solutions; it was obvious that the values were significantly decreased from the first to the sixth profile solution and were slightly decreased around the third model. Moreover, compared with the last three models, the third profile model was easier to accept due to the parsimonious model selection criteria and the principle of interpretability.

**Table 3:** Fit statistics for class models 1 through 6.

Number of profiles	AIC	BIC	a-BIC	Entropy	LMR-LRT	BLRT	Class size, *n* (%)					
1	19916.837	20072.058	19945.159	–	–	–	–	–	–	–	–	–
2	19466.714	19703.426	19509.905	0.756	0.0017	0.0000	227 (63.41)	131(36.59)	–	–	–	–
3	19343.574	19661.778	19401.634	0.847	0.3199	0.0000	27 (7.54)	226 (63.13)	105 (29.33)	–	–	–
4	19214.418	19614.113	19287.347	0.873	0.0828	0.0000	24 (6.70)	219 (61.17)	39 (10.89)	76 (21.23)	–	–
5	19118.961	19600.148	19206.760	0.835	0.3671	0.0000	32 (8.94)	25 (6.98)	73 (20.39)	153 (42.74)	75 (20.95)	–
6	19070.964	19633.642	19173.632	0.820	0.6948	0.0000	32 (8.94)	25 (6.98)	123 (34.36)	60 (16.76)	40 (11.17)	78 (21.79)

AIC, Akaike information criterion; BIC, Bayesian information criterion; a-BIC: adjusted BIC; LMR-LRT, Lo–Mendell–Rubin likelihood ratio test; BLRT, bootstrap likelihood ratio test.

### Profiles characteristics

**Figure 1:**
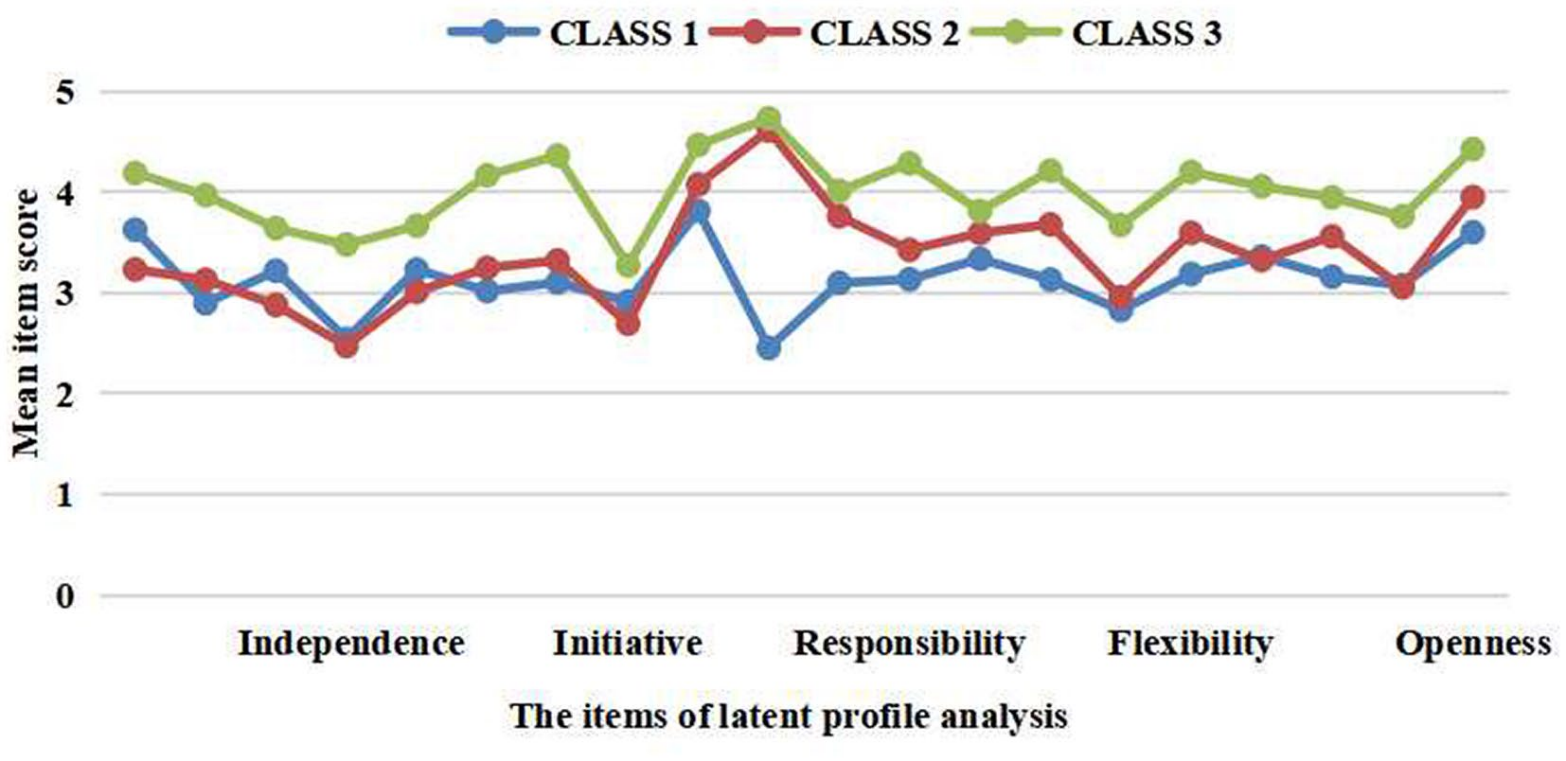
Item means for the third profile mode of interpersonal self-supporting.

Figure 1 shows the plot of the third profile model. The apparent advantage of Class 3 in the scores of interpersonal independence, interpersonal initiative, interpersonal flexibility, and interpersonal openness indicated that Class 3 had high enthusiasm for developing interpersonal relationships, so it was labeled the “ISS-Extrovert” group. Class 2 had the lowest scores on interpersonal independence and interpersonal initiative among these three profiles, indicating that Class 2 had the dependent characteristics of interpersonal communication, and it was labeled the “ISS-Dependent” group. The score gaps of interpersonal responsibility and interpersonal flexibility between Class 1 and the other two groups were more obvious than those of other dimensions, indicating that Class 1 were more individualistic in interpersonal communication, and it was labeled the “ISS-Individualist” group.

According to the results of Shapiro–Wilk tests, One-way ANOVA and the Kruskal–Wallis test were conducted to compare the score of total ISSS-AS and three subscales among the three profiles of ISS. As a whole, the three profiles differed significantly in the interpersonal independence (*χ*^2^ = 110.810, *p* < 0.01), interpersonal initiative (*χ*^2^ = 118.764, *p* < 0.05), interpersonal responsibility (*χ*^2^ = 99.676, *p* < 0.05), interpersonal flexibility (*χ*^2^ = 83.873, *p* < 0.05), interpersonal openness (*χ*^2^ = 58.143, *p* < 0.05) and interpersonal self-supporting (*F* = 265.001, *p* < 0.05). The pairwise comparisons showed that the total ISSS-AS score of ISS-Extrovert group (7.54% of the total sample) was the highest among these three profiles, and that of ISS-Dependent group (63.13% of the total sample) was higher than that of ISS-Individualist group (29.33% of the total sample; *p* < 0.01). Moreover, ISS-Extrovert group had significantly higher score on the five subscales of ISSS-AS than ISS-Dependent group and ISS-Extrovert group (*p* < 0.01). Additionally, there was no statistically significant difference in interpersonal independence, interpersonal initiative, and interpersonal openness between ISS-Individualist group and ISS-Dependent group.

### Sociodemographic characteristics across profiles

Although comparing the sociodemographic characteristics of FNSs among these three profiles was not the main aim of our study, analyzing these differences could be beneficial. Based on the results of the Chi-squared tests, we found no significant difference between these three profiles of ISS in gender (*χ*^2^ = 0.465, *p* = 0.792), age (*χ*^2^ = 0.567, *p* = 0.753), being an only child (*χ*^2^ = 1.921, *p* = 0.383), residence (*χ*^2^ = 0.690, *p* = 0.708), education status of father(*χ*^2^ = 3.938, *p* = 0.415) and mother (*χ*^2^ = 1.229, *p* = 0.873), monthly family income (*χ*^2^ = 5.085, *p* = 0.459), or academic achievement level (*χ*^2^ = 7.774, *p* = 0.100), as presented in Table 1.

### Relationship between identified profiles and PI

The results of BCH tests are shown in Table 4. The three profiles differed significantly in the five subscales of the PIQNS. Regarding pairwise comparisons, the differences in all five dimensions of PIQNS and total PI between ISS-Individualist group and ISS-Extrovert group were significant (*p* < 0.05). ISS-Dependent group and ISS-Extrovert group differed significantly in the three PIQNS dimensions of social comparison and self-reflection, independence of career choice, and social modeling and total PI (*p* < 0.01). However, there were no significant differences between ISS-Individualist group and ISS-Dependent group on the five dimensions of the PIQNS and total PI (*p*>0.05).

**Table 4:** Profile of interpersonal self-support (ISS) membership on professional identity.

	ISS-Individualist (*n* = 27)		ISS-Dependent (*n* = 226)		ISS-Extrovert (*n* = 105)		Overall *χ*^2^	
	*M* (P25, P75)	M/M (SD)	M (P25, P75)	M/M (SD)	*M* (P25, P75)	M/M (SD)		
Professional self-image	17 [14, 18]	16.07	18 [15, 20]	17.74	18 [16, 21]	18.49	6.371*	C1 versus C2 = ns C1 versus C3 = * C2 versus C3 = ns
Benefit of retention and risk of turnover	10 [8, 12]	10.00	11 [9, 12]	10.44	11 [9, 12.5]	10.83	6.158*	C1 versus C2 = ns C1 versus C3 = * C2 versus C3 = ns
Social comparison and self- reflection	11 [9, 12]	10.44	11 [9, 12]	10.64	11 [10, 13]	11.39	20.275 **	C1 versus C2 = ns C1 versus C3 = * C2 versus C3 = **
Independence of career choice	7 [6, 8]	7.26	7 [6, 8]	7.04	8 [7, 8]	7.89	44.399**	C1 versus C2 = ns C1 versus C3 = ** C2 versus C3 = **
Social modeling	7 [5, 7]	6.22	7 [6, 8]	6.68	8 [6, 9]	7.54	26.062**	C1 versus C2 = ns C1 versus C3 = ** C2 versus C3 = **
Professional identity	–	50.393 (1.394)	–	52.419 (0.561)	–	57.448 (1.031)	22.234**	C1 versus C2 = ns C1 versus C3 = ** C2 versus C3 = **

**p < 0.01, *p < 0.05; ns = insignificant.

## Discussion

### Characteristics of ISS and PI

In our study, the FNSs could be classified into three groups: the ISS–Individualist group (29.33% of the total sample), ISS-Dependent group (63.13% of the total sample), and ISS-Extrovert group (7.54% of the total sample). Clearly, more than half of FNSs lacked interpersonal initiative and independence, which indicated that the intention to participate the interpersonal activity was low among students. Prior studies have verified the predictive role of gender in interpersonal initiative and independence (Xia et al., 2012, 2014). In our study, the majority of freshmen were female, and more than half of FNSs were classified into the ISS-Dependent group. A possible explanation for the gender difference in negative affect is that females are more sensitive to interpersonal emotions and events than males due to physiological differences, resulting in psychological distress and hesitation around interpersonal communication (Gao et al., 2020). The differences in self-concept may contribute to the formation of masculinity (such as individualism, fearlessness of death, and confidence) and femininity (such as affection, compassion, and sensitivity to other’s needs; Gao et al., 2020). The results are consistent with a previous finding that males were more likely to be active and independent in the development of interpersonal relationships (Xia et al., 2014). Variable-centered studies focused on evaluating the level of interpersonal communication found that nursing students lacked interpersonal attributes and competence (Mangan et al., 2022). Our person-centered study emphasized the shortage of independence and initiative in communicating with others and expanded our knowledge with respect to the tendencies and characteristics of interpersonal communication among FNSs. As noted above, undergraduate students need to find different kinds of social supports to help them managed unanticipated developments and adjust to the nursing profession (Goodolf, 2018). Freshmen also need more social support in adapting to college life to develop their PI (Goodolf, 2018). The shortage of initiative and independence of ISS is adverse for their future academic and career development, as proactive communication is crucial to both the positive formation of PI and efficient nursing care (Philippa et al., 2021). However, nursing interpersonal communication education has been undervalued in the past (Mangan et al., 2022). Our study highlighted the necessity of increasing nursing students’ confidence in communicating with others.

In the present study, the mean PI score among the FNSs was 3.14 (0.53), which was consistent with the finding of Mao et al. (2021) that the level of PI was moderate among Chinese nursing students. Moreover, they also found that the level of PI declined steadily from the freshmen year to the senior year (Mao et al., 2021). Recently, Nie et al. (2021) showed that the mean score of PI was 3.95 (0.61), which was higher than that of freshman students in our study, using the same measure tool as our study among Chinese senior nursing students with 8 months of experience in clinical nursing rotation training during the coronavirus disease (COVID-19) pandemic. Generally, students will experience the gap between ideal nursing care and complex nursing practice in clinical nursing rotation training (Goodolf, 2018; Mao et al., 2021). Prior studies have indicated that the higher clinical nursing competence promoted by clinical nursing rotation training could not promote the PI of nursing interns (Goodolf, 2018). Nevertheless, apart from personal prior experience, we should not ignore the crucial role of the external environment on PI among FNSs. Undeniably, the outbreak of COVID-19 caused enormous economic losses and life casualties. It also promoted the traditional social image of the nursing group *via* the positive evaluation and description of medical staff by media (Nie et al., 2021). Student nurses in particular were motivated by clinical mentors’ experience fighting the pandemic and recognized the role and value of the nursing profession, which finally promoted their level of PI (Nie et al., 2021). However, personal internal factors are also important for the formation of PI. According to the research of Nie et al. (2021) students with a negative attitude toward the COVID-19 pandemic scored the lowest on PI and highest on intention to leave the nursing profession compared with others.

### Association between ISS and PI

The present study found that the ISS-Individualist group and ISS-Extrovert group were different in all five PIQNS dimensions of professional self-image, benefit of retention and risk of turnover, social comparison and self-reflection, independence of career choice, and social modeling and total PI, while the ISS-Dependent group and ISS-Extrovert group were different in the three PIQNS dimensions of social comparison and self-reflection, independence of career choice, and social modeling and total PI. The ISS-Individualist group had lower levels of interpersonal responsibility and interpersonal flexibility than other two groups. FNSs may adopt negative interpersonal attitudes toward people if their interpersonal responsibility scores were low, resulting in disrespectful behavior in interpersonal activities (Xia et al., 2015). They would not promote their PI by seeking advice from experienced nurses or by initiating a series of social comparison and self-reflection behaviors. Moreover, prior studies have determined that interpersonal responsibility could negatively predict negative effects (Xia et al., 2015; Gong et al., 2017). FNSs with low interpersonal responsibility might be easily affected by unsatisfactory social images of nurses and experience negative professional emotions, resulting in them deciding to leave the nursing profession (Xia et al., 2015; Gong et al., 2017). Furthermore, interpersonal flexibility is an important predictive factor of self-efficacy (Mei et al., 2022). Lower self-efficacy may lead to an unclear professional self-image and difficulties in career decision-making among students (El Othman et al., 2020). However, the levels of interpersonal independence and interpersonal initiative of the ISS-Dependent group were the lowest among the three groups. In other words, FNSs did not initiate communication with successful nurses to elevate their PI by becoming more self-aware and self-reflective (Xia et al., 2015). Many researchers believe that lower interpersonal independence indicates neuroticism, and students with the neuroticism trait were more likely to have a dependent career decision-making style (Xia et al., 2015; Simmonds et al., 2020). Generally, it is difficult for members of the ISS-Individualist and ISS-Dependent groups to achieve positive PI formation. The ISS-Extrovert group, which had significant advantages in the scores of the five dimensions of ISSS-AS, could potentially achieve a better PI.

### Implications for the positive formation of ISS and PI

In our study, a shortage of initiative and independence of ISS was common among Chinese FNSs. Therefore, taking measures to guide the positive formation of ISS to promote the development of PI is necessary for FNSs to adapt to the nursing profession and facilitate positive personal and career development. Regarding the promotion of PI, a scoping review summarized the contributors of the formation of PI addressed in undergraduate nursing pedagogical intervention research, including nursing knowledge and skills, the professional nursing role, beliefs and values, personal attributes, and belongingness (Li et al., 2023). Among these contributors, nursing knowledge and skills was the most commonly cited in diverse pedagogical practice settings (Li et al., 2023). Based on the relationship between PI and ISS, nursing freshmen need more confidence and more general communication knowledge to maintain harmonious social relationships with relatives, friends and faculties. However, interpersonal communication competence is treated as a vital clinical nursing skill, and the relevant courses are set in the senior year in China (Gilligan et al., 2021). More importantly, these courses overemphasize the communication skills, such as empathy, listen, and self-disclosure, while guiding the development of interpersonal independence and initiative is ignored (Gilligan et al., 2021). Regarding the existing characteristics of nursing interpersonal communication curricula, we have several pieces of advice. First, FNSs are in the pre-formal professional education stage, and many nursing professional curricula are not suitable for them (Crawford et al., 2020). Hence, nursing faculties should teach more general communication knowledge *via* workshops or extracurricular activities for FNSs. Second, Crawford et al. (2020) found that paid work increased the interpersonal communication confidence of FNSs, regardless of whether the work was in the health industry or not (Cheung, 2009). More interpersonal practice and simulation activities may encourage freshmen to communicate with others. Finally, nursing teachers may use psychological intervention strategies to promote the formation of PI and ISS among FNSs, such as cognition intervention, group psychological intervention, and empathy intervention (Wang et al., 2022).

In terms of the cultivation of ISS, efforts from both family and school are integral (Xia, 2010). Combining the educational resources of the family and school to promote the development of interpersonal independence and initiative, parent–teacher association may be an option (Fu et al., 2021). Parent–teacher association is one kind of education model that treats the school as the subject and encourages teachers, parents, and students to participate in advancing students’ well-being (Fu et al., 2021). It has been applied in predicting bullying perpetration, assessment of attention deficit/hyperactivity disorder symptoms, and bad behavior correction (Fu et al., 2021; Garcia-Rosales et al., 2021). Based on the career decision making model for college students, which includes the four stages of awareness, planning, commitment, and implementation, we have several pieces of advice (Harren, 1979). First, nursing teachers should construct useful communication channels with parents, such as WeChat group sand QQ groups. Second, at the awareness stage, nursing teachers should evaluate students’ ISS via self-rating and other-rating by parents before they start their college life. Then the feedback on the results of students’ self-rating to parents should be initiated by nursing teachers, synthetically estimating the advantages and weaknesses of students to help them make progress. Afterward, nursing teachers and parents should correct students’ wrong cognition of interpersonal communication before they begin their college life. Third, at the planning and commitment stage, according to the status of correction, personal attributes, family context, and the characteristics of nursing education, nursing teachers, parents, and students should draw up a plan for college life. The potential challenges of adapting to the new environment, new interpersonal relationships, new learning contents, corresponding coping strategies, and social support resources should be identified clearly. Finally, at the implementation stage, nursing teachers should implement activities to encourage freshmen to communicate with others actively and independently.

## Limitations and future directions

This study had several limitations. First, we examined the influencing effect of ISS on PI using a cross-sectional design, which was not suitable for determining the predictive role of ISS. Further studies should try to use a longitudinal design to describe the trajectory of PI and ISS and examine the predictive relationship between these two variables during the whole first year of college life among freshmen. Second, although we used person-centered approach to determine the influencing role of ISS in PI among freshmen, the results could not describe the specific pathway of the influencing relationship. There are two ways to address this shortcoming. On the one hand, further studies should try to describe the evolution of the influencing relationship using qualitative interviews. On the other hand, scholars should construct a hypothetical model based on a theoretical and literature review, and then they should examine the model using a quantitative study design among students. Third, this study recruited freshmen who had already been in college for a semester. In fact, PI is established before beginning college, so future studies should recruit prospective nursing students as a sample. Finally, the freshmen in this study were recruited from only two nursing colleges, and the representativeness and size of the sample were unsatisfactory. Future studies should enrich the sample sources and sample size.

## Conclusion

Previous studies have explored the formation of PI among nursing undergraduates. This study is the first to use a person-centered approach to identify three clusters of individuals with similar characteristics–an ISS-Individualist group, ISS-Dependent group, and ISS-Extrovert group–among Chinese FNSs. We also found that majority of the freshmen were classified in the ISS-Dependent group, and that FNSs with an Extroverted ISS had better PI. Implementing measures to guide the positive formation of Extroverted ISS to promote the development of PI is necessary for FNSs to adapt to the nursing profession and facilitate positive personal and career development. Nursing faculty should attempt to consider psychological interventions and organize workshops, extracurricular activities, interpersonal practice, and simulation activities to facilitate initiative and independence in interpersonal communication, and thus promote the formation of PI and ISS among FNSs. Furthermore, nursing faculties should evaluate the FNSs’ ISS and provide feedback on the results to the students’ parents via useful communication channels.

## Data availability statement

The raw data supporting the conclusions of this article will be made available by the authors, without undue reservation.

## Ethics statement

The studies involving human participants were reviewed and approved by Ethics Committee of the Anhui Medical University (approval number 20210075). Written informed consent to participate in this study was provided by the participants’ legal guardian/next of kin.

## Author contributions

TZ and DS: study conception and design and critical revision of the article. SL and YY: data collection. TZ: data analysis, interpretation, and drafting of the manuscript. All authors contributed to the article and approved the submitted version.

## Funding

This work was supported by the University Natural Science Research Project of Anhui Province (approval number: KJ2021A0256), the Natural Science Research Project of School of Nursing of Anhui Medical University (approval number: hlpy20210019), and the Anhui Provincial Department of Education Quality Engineering Project (approval numbers: 2020kcszyjxm124 and 2021jyxm0672).

## Conflict of interest

The authors declare that the research was conducted in the absence of any commercial or financial relationships that could be construed as a potential conflict of interest.

## Publisher’s note

All claims expressed in this article are solely those of the authors and do not necessarily represent those of their affiliated organizations, or those of the publisher, the editors and the reviewers. Any product that may be evaluated in this article, or claim that may be made by its manufacturer, is not guaranteed or endorsed by the publisher.

## Supplementary material

The Supplementary material for this article can be found online at: https://www.frontiersin.org/articles/10.3389/fpsyg.2023.1123625/full#supplementary-material
