# Supplementary material for: Does interpersonal self-support matter for freshman nursing students’ professional identity? Evidence from mainland China
Source: Front Psychol. 2023 May 23;14:1123625. doi: 10.3389/fpsyg.2023.1123625 (PMC10243468; doi:10.3389/fpsyg.2023.1123625)
Supplement: Supplementary file 1 [file Table_1.docx]

Supplementary Table S1 The Interpersonal Self-Support Scale for Adolescent Students

Instruction: “Please evaluate how much you disagree or agree with the following statements.” (1=Strongly Disagree, 2=Disagree, 3=Neutral, 4=Agree, 5= Strongly Agree).

| **Items** | **Strongly Disagree** | **Disagree** | **Neutral** | **Agree** | **Strongly Agree** |
| --- | --- | --- | --- | --- | --- |
| 1.It is not easy for me to talk with strangers by myself. | 1 | 2 | 3 | 4 | 5 |
| 2.Most of my classmates are more actively than me in communication with others. | 1 | 2 | 3 | 4 | 5 |
| 3.I always make promises carelessly. | 1 | 2 | 3 | 4 | 5 |
| 4.I am good at helping others get out of embarrassing situation in social interactions. | 1 | 2 | 3 | 4 | 5 |
| 5.I cannot accept the one whose personality is quite different from me. | 1 | 2 | 3 | 4 | 5 |
| 6.I do not dare to visit other houses by myself. | 1 | 2 | 3 | 4 | 5 |
| 7.I actively make new friends. | 1 | 2 | 3 | 4 | 5 |
| 8.I cannot keep secrets of my friend(s) from others. | 1 | 2 | 3 | 4 | 5 |
| 9.I am good at reconciling different opinions. | 1 | 2 | 3 | 4 | 5 |
| 10.I dislike those who behave differently. | 1 | 2 | 3 | 4 | 5 |
| 11.I feel nervous when I communicate with opposite sex by myself. | 1 | 2 | 3 | 4 | 5 |
| 12.I do not communicate with others unless they initiatively talk with me at first. | 1 | 2 | 3 | 4 | 5 |
| 13.I never give others empty promises. | 1 | 2 | 3 | 4 | 5 |
| 14.When I turn down the request of others, I can make them not feel offended. | 1 | 2 | 3 | 4 | 5 |
| 15.I cannot accept those who have many shortcomings. | 1 | 2 | 3 | 4 | 5 |
| 16.I feel nervous in a situation where strangers are around me. | 1 | 2 | 3 | 4 | 5 |
| 17.I usually introduce myself firstly when I meet someone at the first time. | 1 | 2 | 3 | 4 | 5 |
| 18.Once getting into trouble, I want to give up my promises. | 1 | 2 | 3 | 4 | 5 |
| 19.I am good at making both sides feel respected and cared in interpersonal communication. | 1 | 2 | 3 | 4 | 5 |
| 20.I believe that annoying persons should be isolated. | 1 | 2 | 3 | 4 | 5 |

Supplementary Table S2 The Professional Identity Questionnaire for Nurse Students

Instruction: “Please evaluate how much you disagree or agree with the following statements.” (1=Strongly Disagree, 2=Disagree, 3=Neutral, 4=Agree, 5= Strongly Agree).

| **Items** | **Strongly Disagree** | **Disagree** | **Neutral** | **Agree** | **Strongly Agree** |
| --- | --- | --- | --- | --- | --- |
| 1.I like to be a nurse. | 1 | 2 | 4 | 5 | 6 |
| 2.I like to know more professional developing stories of some successful persons in nursing field. | 1 | 2 | 4 | 5 | 6 |
| 3.I like to communicate with predecessors of nursing field. | 1 | 2 | 4 | 5 | 6 |
| 4.I will choose the job I like no matter what other person say. | 1 | 2 | 4 | 5 | 6 |
| 5.I have already devote too much to nursing career including economic aspect and effort, so I don’t want to leave the field of nursing. | 1 | 2 | 4 | 5 | 6 |
| 6.I have no plan to change my career direction. | 1 | 2 | 4 | 5 | 6 |
| 7.I often explore my professional development by reflecting my interest, personality and values on myself. | 1 | 2 | 4 | 5 | 6 |
| 8.Being a nurse make good use of my competence and advantage. | 1 | 2 | 4 | 5 | 6 |
| 9.I like my major and am positively preparing for my future work. | 1 | 2 | 4 | 5 | 6 |
| 10.Turnover will cause me emotional wounds. | 1 | 2 | 4 | 5 | 6 |
| 11.I am proud of working in the field of nursing. | 1 | 2 | 4 | 5 | 6 |
| 12.My knowledge about nursing career mainly came from parents, teacher and other relatives. | 1 | 2 | 4 | 5 | 6 |
| 13.I try to know the condition of other career field so that I can make my professional belief more strong. | 1 | 2 | 4 | 5 | 6 |
| 14.Being a nurse can bring my creativity into play. | 1 | 2 | 4 | 5 | 6 |
| 15.Both one’s own ideal and surrounding factors should be considered during the process of career choice. | 1 | 2 | 4 | 5 | 6 |
| 16.Being a nurse makes me happy. | 1 | 2 | 4 | 5 | 6 |
| 17.I am sure I will success in nursing field. | 1 | 2 | 4 | 5 | 6 |
